# Latent Class‐Specific Psychosocial and Behavioral Determinants of Preventive Behavioral Intentions for Emerging Infectious Diseases Among Korean Adults: A Mixture Regression Model Approach

**DOI:** 10.1111/phn.70105

**Published:** 2026-02-25

**Authors:** Yong‐Hyun Moon, Jihea Choi

**Affiliations:** ^1^ Department of Nursing Yonsei University Wonju College of Nursing Wonju Republic of Korea

**Keywords:** awareness, emerging infectious diseases, intentions, regression analysis, self‐efficacy, social responsibility, vaccination

## Abstract

**Objective:**

This study aimed to identify latent psychosocial–behavioral subgroups among Korean adults and examine how emerging infectious disease (EID) awareness, social responsibility, self‐efficacy, vaccination behavior, and mask‐wearing behavior can predict preventive behavioral intentions.

**Design and Sample:**

A cross‐sectional survey was conducted with 149 Korean adults aged ≥19 years and recruited through convenience sampling from two metropolitan areas.

**Measurements:**

Participants completed structured questionnaires assessing psychosocial (EID awareness, social responsibility, and self‐efficacy) and behavioral (vaccination and mask‐wearing behavior) variables.

**Results:**

Overall, conventional regression analysis showed that social responsibility and vaccination behavior significantly predicted preventive behavioral intentions for EIDs. Mixture regression analysis revealed two latent classes. In Class 1, preventive intentions were influenced by awareness, social responsibility, vaccination, mask‐wearing, age, and COVID‐19 history. In Class 2, self‐efficacy, social responsibility, and vaccination behavior were significant predictors, with COVID‐19 history negatively associated. These class‐specific patterns in awareness and behavior underscore the importance of subgroup analysis.

**Conclusions:**

Psychosocial and behavioral predictors of preventive behavioral intentions differ across subgroups. These findings emphasize the need for targeted public health strategies that account for population heterogeneity. Moreover, tailored nursing interventions can enhance the effectiveness of EID prevention efforts in diverse communities.

## 1 Introduction

Emerging infectious diseases (EIDs), including severe acute respiratory syndrome (SARS), Middle East respiratory syndrome (MERS), and coronavirus disease 2019 (COVID‐19), have repeatedly challenged global health systems over the past two decades, underscoring the importance of institutional preparedness and the critical role of individual‐level preventive behavior (Jones et al. 2008; Klusowski et al. 2019; Peeri et al. 2020; You et al. 2024). During the COVID‐19 pandemic, South Korea garnered international attention for its exceptionally high adherence to preventive measures, particularly mask‐wearing and vaccination (Kim et al. 2022; Kim et al. 2021). However, as mandated measures have lessened, sustaining preventive behavioral intentions for future EID threats increasingly relies on individual psychosocial and behavioral characteristics.

EID awareness, social responsibility, and self‐efficacy were identified as key psychosocial predictors of preventive behavior (Luo et al. 2020; McElfish et al. 2021; Pak 2021). Moreover, prior behavioral patterns, such as past vaccination uptake and habitual mask‐wearing, have influenced future behavioral intentions (Abroms et al. 2024; Murakami et al. 2025). These findings are supported by recent literature reviews and meta‐analytic studies, which demonstrated that disease awareness, perceived risk, social norms, and self‐efficacy consistently predict preventive intentions and behaviors across diverse populations during infectious disease outbreaks (Betsch et al. 2018; Brooks et al. 2020; Chew et al. 2020; Schumpe et al. 2022). Specifically, motivational factors such as self‐efficacy and collective responsibility appear to help sustain preventive behaviors when formal public health mandates are relaxed.

Regardless, many studies have treated populations as homogeneous without adequately considering latent heterogeneity in psychosocial and behavioral profiles. Importantly, existing reviews report substantial heterogeneity in effect sizes across studies; thus, psychosocial determinants of preventive behavioral intentions might not have operated uniformly across individuals. However, most prior syntheses rely on population‐averaged analytic approaches, offering limited insight into unobserved subgroups with distinct psychosocial–behavioral profiles (Chew et al. 2020; Wang et al. 2024). From a public health nursing perspective, this represents a critical gap, considering that tailored, subgroup‐sensitive interventions are generally more effective than generalized strategies, particularly in high‐compliance contexts.

To address this gap, this study applies mixture regression modeling to identify latent psychosocial–behavioral subgroups among Korean adults and examine how psychosocial characteristics (EID awareness, social responsibility, and self‐efficacy) and behavioral characteristics (past vaccination behavior and mask‐wearing behavior) jointly predict preventive behavioral intentions for EIDs across the overall population and latent classes (Chew et al. 2020; Guilamo‐Ramos et al. 2021; Wang et al. 2024). Understanding the heterogeneity in public health behavior influences designing targeted interventions, as traditional regression models may obscure important subgroup differences. This study by using mixture regression, aimed to uncover latent subpopulations and evaluate whether key predictors exert differential effects across these classes (Klusowski et al. 2019; You et al. 2024), ultimately providing evidence to guide the development of tailored nursing interventions and public health strategies. Accordingly, examining latent psychosocial–behavioral subgroups is useful for capturing heterogeneity in preventive behavioral intentions and for informing more nuanced nursing and public health responses to future EID threats beyond COVID‐19.

## 2 Background

The complexity of preventive behavioral intentions for EIDs stems not only in individual psychological dispositions but also in their past behavioral experiences (Brooks et al. 2020; Kim and Kim 2020; McElfish et al. 2021). EID awareness equips individuals with knowledge about transmission risks (Dashti et al. 2022; Pak 2021), social responsibility (Ali et al. 2021; Kim et al. 2022) promotes communal motivations for health‐protective actions, and self‐efficacy boosts confidence in individuals’ ability to carry out preventive behaviors (McElfish et al. 2021; Schumpe et al. 2022). Collectively, these psychosocial factors interact with prior behaviors, including vaccination uptake and mask‐wearing, to shape preventive behavioral intentions dynamically rather than through single, isolated pathways.

Conventional regression models often assume population homogeneity, which limits their ability to detect subgroup‐specific variations among predictors (Bouwmeester et al. 2004; Ding 2006; Jaki et al. 2019). Conversely, mixture regression modeling is a person‐centered analytic approach that identifies unobserved subgroups (latent classes) within a population and allows regression coefficients to vary across these classes (Ding 2006; You et al. 2024). It enables a more nuanced examination of how psychosocial and behavioral determinants function differently across distinct subpopulations, thereby capturing heterogeneity, which may be obscured in population‐averaged analyses (Klusowski et al. 2019).

According to recent methodological advances, robust estimation strategies are important in mixture models to ensure reliable subgroup detection, specifically in heterogeneous samples (You et al. 2024). In Korea, where public compliance with preventive measures is generally high, latent heterogeneity in psychosocial motivation and behavioral patterns may be less visible at the aggregate level but remains substantively meaningful (Ali et al. 2021; Kim et al. 2022). Therefore, applying mixture regression modeling provides an appropriate approach for identifying subgroup‐specific determinants of preventive behavioral intentions and for informing more precise, evidence‐based nursing interventions and public health strategies.

## 3 Methods

### 3.1 Study Design

This cross‐sectional quantitative study was conducted to identify latent psychosocial–behavioral subgroups among adults and examine how psychosocial (EID awareness, social responsibility, and self‐efficacy) and behavioral (vaccination and mask‐wearing behaviors) characteristics predict preventive behavioral intentions for EIDs.

### 3.2 Participants

A convenience sampling strategy was used to recruit adults aged ≥19 years in South Korea. Individuals who understood the study's purpose and procedures and provided written informed consent were included. The required sample size was calculated using G*Power 3.1.9.7 (Düsseldorf, Germany) for regression analysis, with parameters set to a medium effect size (*f^2^*) of 0.15, significance level of 0.05, power of 0.95, and 10 independent variables, yielding at least 146 participants. To accommodate an anticipated 5% nonresponse rate, 153 individuals were recruited. After excluding incomplete or invalid responses, the final analysis includes 149 participants (response rate, 97.4%).

### 3.3 Data Collection

Data were collected between April 6 and April 12, 2023, from two purposively selected metropolitan areas (S and W cities) in South Korea, which were chosen for their accessibility and population density. Participants were recruited through random on‐site strategies in high‐traffic public locations, such as public transportation terminals, government office waiting areas, parks, and large commercial centers. The study's purpose, procedures, and significance directly were explained to each potential participant, and only those who voluntarily provided written informed consent were considered. They completed a self‐administered questionnaire in a designated area, with the researcher available to provide clarification as needed following standardized procedures. Each survey was completed 10–15 min. Completed questionnaires were checked on‐site for completeness, sealed in prepared envelopes, and collected by the researcher. All data collection procedures adhered to ethical guidelines and ensured voluntary participation, anonymity, and confidentiality.

### 3.4 Ethical Considerations

This was approved by the institutional review board of the Yonsei University Hospital Research Review Committee (IRB Approval No. CR322159). All participants received detailed information regarding the study's purpose, procedures, voluntary nature of participation, and confidentiality of their responses. All participants provided written informed consent before data collection. Anonymity was ensured by excluding any personally identifiable information, and the collected data were securely stored and used solely for research. The data will be retained for 3 years following the completion of the study.

### 3.5 Measures

A structured self‐report questionnaire was administered to assess participants’ demographic characteristics; EID awareness, social responsibility, and self‐efficacy as psychosocial characteristics; vaccination and mask‐wearing behaviors as behavioral characteristics; and preventive behavioral intention related to EIDs. To reduce potential bias, validated measurement tools were used, and participants were instructed to complete the questionnaires anonymously to minimize social desirability bias.

#### 3.5.1 Demographic Characteristics

Demographic characteristics, including age, sex, number of family members, and history of COVID‐19, were assessed.

#### 3.5.2 EID Awareness

EID awareness was defined as the extent to which individuals subjectively discern and evaluate information about EIDs. In this study, a 10‐item awareness scale developed in Korean was employed (Park and Lee 2019), rated on a 5‐point Likert scale (1 = strongly disagree, 5 = strongly agree), and higher mean scores indicate greater awareness. Although the original tool gained a relatively low internal consistency (Kuder–Richardson Formula 20 [KR‐20] = 0.59), it was selected for this study owing to the lack of validated EID awareness measures for the general population and its relevance to the pandemic setting. In this study, this scale had a KR‐20 of 0.62.

#### 3.5.3 Social Responsibility

Social responsibility was defined as an individual's attitude of interest and obligation toward social issues, leading to behaviors that contribute to society. The social responsibility scale, which was originally developed by Conrad and Hedin (2008) and adapted to the Korean context by Lim (2006), was employed. The tool consists of 27 items across five subdomains: responsibility attitude (5 items), responsibility obligation (5 items), responsibility competence (3 items), responsibility efficacy (7 items), and responsibility performance (7 items). Each item is rated on a 5‐point Likert scale (1 = strongly disagree, 5 = strongly agree), and the mean scores were used in this study. Higher scores indicate greater social responsibility. Cronbach's α for this tool was 0.70 in Conrad and Hedin (2008) and was 0.89 in the present study.

#### 3.5.4 Self‐Efficacy

Self‐efficacy was defined as an individual's belief and confidence in one's ability to successfully perform specific tasks. This study utilized the self‐efficacy scale originally developed by Sherer et al. (1982) and reconstructed by Oh et al. (1997) for the Korean context. This scale includes 13 items, each rated on a 5‐point Likert scale (1 = strongly disagree, 5 = strongly agree), and mean scores were used for the analysis. Higher scores indicate greater self‐efficacy. Cronbach's α for this tool was 0.83 in Oh et al. (1997) and 0.87 in the present study.

#### 3.5.5 Vaccination Behavior

Vaccination behavior was defined as receiving COVID‐19 vaccination, classified as a legally designated EID vaccination in South Korea. During data collection (April 2023), South Korea was following the COVID‐19 vaccination guidelines by the Central Disease Control Headquarters under Korea Disease Control and Prevention Agency (KDCA) revised on January 30, 2023 (Korea Disease Control and Prevention Agency, 2023a). These guidelines recommended additional booster doses (third and fourth doses) for individuals aged ≥12 years who had completed the first and second doses (primary series), with particular emphasis on completing the fourth dose for older adults and residents of high‐risk facilities. Accordingly, vaccination behavior was assessed using a self‐reported item asking participants to indicate the number of COVID‐19 vaccine doses they had received. Responses were coded as follows: 0, none; 1, first dose; 2, second dose; 3, third dose; and 4, fourth dose, with higher scores indicating greater adherence to the recommended vaccination behavior.

#### 3.5.6 Mask‐Wearing Behavior

Mask‐wearing behavior was assessed using a self‐reported item assessing the frequency of mask‐wearing in public spaces. During data collection, South Korea was adhering to updated guidelines issued by the KDCA regarding the downgraded COVID‐19 crisis level, as the 8^th^ edition revised on March 20, 2023 (Korea Disease Control and Prevention Agency, 2023b). Under these revised guidelines, mask‐wearing mandates were significantly relaxed compared with that in the 7th edition. Specifically, mandatory mask use was limited to high‐risk residential facilities, medical institutions, and pharmacies, whereas previous mandates included indoor settings on public transportation. Accordingly, responses were rated on a 3‐point scale (1 = rarely, 2 = sometimes, 3 = mostly), and higher scores indicate more consistent mask‐wearing behavior.

#### 3.5.7 Preventive Behavioral Intentions for EIDs

An individual's will to engage in practices that prevent new infectious disease spread indicated preventive behavioral intentions for EIDs. This study used a 5‐item scale originally developed by Kim (2022) and later adopted by Lee (2022). Each item was rated on a 5‐point Likert scale (1 = not at all, 2 = no, 3 = neutral, 4 = yes, 5 = very much yes); the higher the total score, the stronger the preventive behavioral intentions. Its Cronbach's α coefficient was 0.89 in the study by Lee (2022) and 0.83 in the present study.

### 3.6 Data Analysis

All data were analyzed using IBM SPSS Statistics for Windows version 27 (IBM Corp., Armonk, NY, USA) and Python version 3.11.12 (Python Software Foundation, Wilmington, DE, USA). Descriptive statistics, including frequencies, percentages, means, and standard deviations, were used to summarize participants’ demographic characteristics and study variables. Cronbach's α coefficients were calculated to assess the internal consistency of multi‐item scales.

The predictors of preventive behavioral intentions for EIDs were examined via conventional regression analysis and mixture regression modeling. Mixture regression modeling is a person‐centered analytic approach that identifies unobserved subgroups within a population and allows regression coefficients to vary across latent classes, thereby addressing population heterogeneity that may be overlooked in traditional regression models (Ding 2006; Klusowski et al. 2019; You et al. 2024). The present study estimated and compared models that had varying numbers of latent classes to determine whether the relationships between psychosocial and behavioral factors and preventive behavioral intentions differed across subgroups.

Model selection was based on standard fit indices, including the Akaike information criterion (AIC), Bayesian information criterion (BIC), log‐likelihood values, and adjusted *R^2^*. The optimal model was identified by considering overall model fit and parsimony. Standardized regression coefficients were calculated to facilitate comparisons across latent classes, and Wald tests were used to evaluate between‐class differences in predictor effects. All statistical tests were two‐tailed, with significance levels set at *p* < 0.1, *p* < 0.05, and *p* < 0.001.

## 4 Results

### 4.1 Characteristics of the Participants

A total of 149 participants were included, with a mean age of 36.50 (SD = 9.81; range = 23–64) years. Among the participants, 57.7% were male, and 42.3% were female; 70.5% lived with at least one other family member, and 21.5% reported a history of COVID‐19. The mean scores of psychosocial variables were 3.29 (SD = 0.40), 3.40 (SD = 0.42), and 3.58 (SD = 0.50) for EID awareness, social responsibility, and self‐efficacy, respectively. The mean scores of behavioral variables were 2.63 (SD = 0.68) and 2.78 (SD = 0.48) for vaccination and mask‐wearing behaviors, respectively. The mean preventive behavioral intention score was 3.31 (SD = 0.75) (Table 1).

**TABLE 1:** Participant characteristics (*N* = 149).

	Variables	Categories	*n* (%) or Mean ± SD	Range
Demographic characteristics	Age (years)		36.50 ± 9.81	23–64
	Sex	Male	86 (57.7)	
		Female	63 (42.3)	
	Number of family members	1	44 (29.5)	
		≥2	105 (70.5)	
	COVID‐19 confirmed history	No	117 (78.5)	
		Yes	32 (21.5)	
Psychosocial characteristics	EID awareness		3.29 ± 0.40	2.00–4.10
	Social responsibility		3.40 ± 0.42	2.26–4.56
	Self‐efficacy		3.58 ± 0.50	2.38–4.69
Behavioral characteristics	Vaccination behavior		2.63 ± 0.68	0–4
	Mask‐wearing behavior		2.78 ± 0.48	1–3
Preventive behavioral intention for EIDs			3.31 ± 0.75	1.10–5.00

Abbreviations: COVID‐19, coronavirus disease 2019; EIDs, emerging infectious diseases; SD, standard deviation.

### 4.2 Model Fit Verification According to the Number of Latent Classes

Model fit was assessed to determine the optimal number of latent classes (Table 2). Compared with the one‐class model as a conventional regression model (adjusted *R^2^* = 0.344, AIC = 372.65, BIC = 405.70), the two‐class model as a mixture regression model revealed substantially improved fit indices, with a higher adjusted *R^2^* (0.597) and lower AIC (165.90) and BIC (234.99). The log‐likelihood also improved notably (ΔLL = 115.38), which indicates a better model fit. These results suggest that the two‐class model offers a superior explanation of the data compared with the one‐class model.

**TABLE 2:** Model fit verification according to the number of latent classes (*N* = 149).

# of Latent classes	Adj_*R^2^*	LL	AIC	BIC	Δ Adj_*R^2^*	Δ LL	Δ AIC	Δ BIC
1	0.344	−175.33	372.65	405.70				
2	0.597	−59.95	165.90	234.99	0.25	115.38	−206.75	−170.70

Abbreviations: Adj_*R^2^*, Adjusted *R^2^*; AIC, Akaike information criterion; BIC, Bayesian information criterion; LL, log‐likelihood; Δ, change from the 1‐class model.

### 4.3 Comparison of Predictor Effects Between Conventional and Mixture Regression Models

Table 3 compares the regression coefficient estimates between the conventional and mixture regression models, focusing on differences between the two latent classes.

**TABLE 3:** Comparison of predictor effects between conventional and mixture regression models (*N* = 149).

Variables	Conventional regression model	Mixture regression models		
	Full sample (*n* = 149)	Latent class 1 (*n* = 92)	Latent class 2 (*n* = 57)	Wald *χ^2^*
	*β* (SE)	*β* (SE)		
Intercept	0.000 (0.067)	0.046 (0.050)	−0.030 (0.100)	0.457
Psychosocial and behavioral determinants				
EID awareness	0.118 (0.078)	0.351 (0.056) ***	−0.170 (0.124)	14.660 ***
Social responsibility	0.432 (0.089) ***	0.320 (0.065) ***	0.571 (0.134) ***	2.778 *
Self‐efficacy	0.130 (0.079)	0.043 (0.061)	0.207 (0.113) *	1.616
Vaccination behavior	0.184 (0.069) **	0.121 (0.053) **	0.208 (0.101) **	0.582
Mask‐wearing behavior	0.037 (0.071)	0.279 (0.055) ***	−0.119 (0.101)	11.822 ***
Covariates				
Age	0.077 (0.071)	0.090 (0.052) *	0.077 (0.110)	0.012
Sex	0.005 (0.070)	−0.047 (0.054)	0.038 (0.104)	0.532
Number of family members	−0.057 (0.070)	−0.064 (0.054)	−0.065 (0.102)	0.001
COVID‐19 confirmed history	0.035 (0.069)	0.291 (0.052) ***	−0.242 (0.103) **	21.497 ***
*R^2^* (Adj_*R^2^*)	0.384 (0.344)	0.767(0.735)	0.401(0.316)	

*Note*: *p < 0.1; **p < 0.05; ***p < 0.001.

Abbreviations: Adj_R^2^, Adjusted R^2^; β, standardized regression coefficient; COVID‐19, coronavirus disease 2019; EIDs, emerging infectious diseases; SE, standard error; Wald, Wald chi‐square statistic for between‐class differences.

For the conventional regression model applied to the full sample (*n* = 149), social responsibility (*β* = 0.432, *p* < 0.001) and vaccination behavior (*β* = 0.184, *p* < 0.05) emerged as significant predictors of preventive behavioral intentions for EIDs.

The mixture regression approach revealed distinct subgroup patterns. In latent class 1 (*n* = 92), a broader set of predictors significantly influenced preventive behavioral intentions: EID awareness (*β* = 0.351, *p* < 0.001), social responsibility (*β* = 0.320, *p* < 0.001), vaccination behavior (*β* = 0.121, *p* < 0.001), mask‐wearing behavior (*β* = 0.279, *p* < 0.001), age (*β* = 0.090, *p* < 0.1), and confirmed COVID‐19 history (*β* = 0.291, *p* < 0.001). Notably, awareness was strongly and positively associated with the outcome in latent class 1 but showed a nonsignificant negative association in latent class 2 (*β* = −0.160, *p* = 0.170). In latent class 2 (*n* = 57), social responsibility (*β* = 0.571, *p* < 0.001), self‐efficacy (*β* = 0.207, *p* < 0.1), and vaccination behavior (*β* = 0.208, *p* < 0.05) were significant positive predictors, whereas confirmed COVID‐19 history had a significant negative effect (β = −0.242, *p* < 0.05). Mask‐wearing behavior, which was a strong positive predictor in class 1, was not significant in class 2. Age and sex showed no significant effects in either class.

Wald tests confirmed significant between‐class differences in the effects of awareness (*χ^2^* = 14.66, *p* < 0.001), mask‐wearing behavior (*χ^2^* = 11.822, *p* < 0.001), and confirmed COVID‐19 history (*χ^2^* = 21.497, *p* < 0.001), indicating that these predictors influence preventive behavioral intentions in a class‐dependent manner. Although social responsibility was a significant positive predictor in both classes, a marginal Wald result (*χ^2^* = 2.778, *p* < 0.1) indicated potential variations in the strength of social norms across groups. Self‐efficacy showed marginal significance in class 2 (*p* < 0.1) but without significant difference between‐class.

### 4.4 Characteristics of Latent Classes

Table 4 shows the differences between the two latent classes. Latent classes 1 (61.7%) and 2 (38.3%) demonstrated comparable mean ages (36.22 vs. 36.96 years) and sex distributions (male, 56.5% vs. 59.6%). However, latent class 1 included more participants living with two or more family members (72.8%) but fewer participants with a history of COVID‐19 (20.7%) compared with latent class 2 (66.7% and 22.8%, respectively).

**TABLE 4:** Characteristics of latent classes (*N* = 149).

Variables		Latent class 1	Latent class 2
		*n* (%) or Mean ± SD	
Sample size (%)		92 (61.7)	57 (38.3)
Psychosocial and behavioral determinants			
EID awareness		3.33 ± 0.42	3.24 ± 0.37
Social responsibility		3.41 ± 0.41	3.37 ± 0.42
Self‐efficacy		3.59 ± 0.48	3.55 ± 0.52
Vaccination behavior		2.65 ± 0.67	2.60 ± 0.70
Mask‐wearing behavior		3.40 ± 0.47	3.18 ± 0.48
Covariates			
Age		36.22 ± 9.91	36.96 ± 9.53
Sex	Male	52 (56.5)	34 (59.6)
	Female	40 (43.5)	23 (40.4)
Number of family members	1	25 (27.2)	19 (33.3)
	≥2	67 (72.8)	38 (66.7)
COVID‐19 confirmed history	No	73 (79.3)	44 (77.2)
	Yes	19 (20.7)	13 (22.8)

Abbreviations: COVID‐19, coronavirus disease 2019; EIDs, emerging infectious diseases; SD, standard deviation.

Regarding psychosocial and behavioral characteristics, latent class 1 reported slightly higher EID awareness, social responsibility, and mask‐wearing behavior, whereas latent class 2 showed slightly higher self‐efficacy and vaccination behavior. These distinctions indicate nuanced subgroup differences relative to the overall sample as a conventional regression model.

## 5 Discussion

This study demonstrated that the psychosocial and behavioral determinants of preventive behavioral intentions for EIDs operate differently across latent subgroups involving Korean adults. Although conventional regression analysis identified social responsibility and vaccination behavior as primary predictors at the population level, mixture regression modeling revealed two distinct latent classes with different psychosocial and behavioral patterns (Ding 2006; You et al. 2024). By considering hidden subgroup‐specific effects, this study overcomes a key limitation of population‐averaged models and provides a more precise empirical basis for targeted public health nursing and prevention strategies (Jaki et al. 2019; Klusowski et al. 2019).

Latent class 1 was characterized by a broad range of predictors, including EID awareness, social responsibility, vaccination behavior, mask‐wearing behavior, age, and COVID‐19 history. In these individuals, cognitive awareness and social responsibility synergistically strengthened behavioral intentions, supported by habitual adherence behaviors such as mask‐wearing (Luo et al. 2020; Pak 2021). This finding aligns with the results of previous studies showing that collectivist cultures, such as South Korea, emphasize communal values and social harmony, which significantly boost public compliance during health crises (Kim et al. 2022; McElfish et al. 2021). Notably, awareness of infectious disease risk is widely associated with increased risk appraisal and protective behavior, as demonstrated in studies examining responses to MERS, SARS, and COVID‐19 (Ali et al. 2021; Pak 2021).

Conversely, latent class 2 exhibited a motivational profile focused on personal efficacy, with self‐efficacy standing out as a key predictor along with social responsibility and vaccination behavior, whereas awareness and mask‐wearing were not significant factors. This pattern indicates that individual agency and confidence in one's ability to perform preventive actions significantly influence driving intentions in this subgroup (Varasteh et al. 2022). Interestingly, COVID‐19 history in class 2 was negatively associated with preventive intention, which potentially reflects behavioral fatigue or a belief in acquired immunity, a similar finding in some Asian and Middle Eastern contexts but contrasted Western studies where prior infection often increases future compliance because of increased risk perception (Murakami et al. 2025).

The study presents further evidence that key psychological constructs such as awareness, perceived threat, and habitual behavior function differently across unobserved subgroups. Despite prior exposure or widespread public health messaging, the differential response to mask‐wearing and confirmed infection history indicates that some individuals may resist behavioral interventions (Brooks et al. 2020; Schumpe et al. 2022). Importantly, the non‐significance of demographic variables, such as age and sex across both classes, emphasizes the need for interventions to focus on modifiable psychological and behavioral traits, a direction supported by international behavioral science research (Betsch et al. 2018).

These results have important implications for public health strategy. Generalized, one‐size‐fits‐all interventions may be inadequate to achieve optimal compliance in heterogeneous populations. For individuals aligned with latent class 1, public health initiatives should reinforce communal motivation, raise disease awareness, and sustain normative behaviors such as mask‐wearing. Conversely, for latent class 2, interventions should prioritize promoting self‐efficacy, delivering personalized behavioral support, and addressing misconceptions about immunity after infection. Tailoring strategies to fit subgroup‐specific drivers is important for strengthening preparedness and responsiveness to threats of future EIDs.

Methodologically, this study presents the added value of mixture regression modeling in public health research. By moving beyond conventional regression frameworks allows for a deeper comprehension of subgroup‐specific predictors, aligning with calls in behavioral health science to account for population heterogeneity when developing interventions (Ding 2006; Jaki et al. 2019; You et al. 2024). This approach is salient in high‐compliance societies, such as Korea (Kim et al. 2022), where apparent surface‐level uniformity may conceal motivational diversity and behavioral vulnerabilities.

Future longitudinal study studies should build on these insights to capture the evolution of subgroup‐specific behavioral patterns over time and assess their stability under changing public health mandates. Moreover, comparative cross‐national studies could illuminate how differences in cultural, political, and healthcare system shape the complex interplay between psychosocial and behavioral determinants across diverse global populations. Expanding the application of mixture models and other advanced statistical techniques will reinforce the empirical basis for designing precise, evidence‐based nursing, and public health interventions that can meet the challenges posed by future infectious disease threats.

### 5.1 Study Limitations

This study has several limitations. First, the cross‐sectional design precludes causal inferences regarding the relationships between psychosocial and behavioral factors and preventive behavioral intentions for EIDs. Thus, to confirm the temporal and directional nature of these associations, longitudinal studies are needed. Second, all study variables were measured using self‐reported questionnaires, thereby possibly introducing measurement bias, including recall bias and social desirability bias. Such concerns were mitigated using validated instruments, and data were collected anonymously to reduce response distortion. Nevertheless, residual measurement bias cannot be fully excluded. Third, although the sample size met the minimum requirement for regression analysis, the overall sample is relatively small, and the latent classes have unequal sizes, making the findings less generalizable. In particular, the statistical power to detect minor subgroup‐specific effects may have been reduced because of latent class 2's smaller size. However, mixture regression modeling is specifically designed to explore population heterogeneity in exploratory research settings, and the substantial improvement in model fit indices supports the robustness of the identified latent class solution. Further researches with larger and more diverse samples are warranted to validate the stability of these subgroup patterns and enhance generalizability.

### 5.2 Implications for Public Health Nursing

The results of this study highlight the importance of developing evidence‐based, tailored nursing interventions that integrate the heterogeneity of psychosocial and behavioral determinants observed during the COVID‐19 pandemic. Rather than applying generalized strategies, nursing practice should adopt precise, subgroup‐specific approaches based on psychosocial subgroups to improve the relevance and effectiveness of preventive interventions. Specifically, nurses should systematically assess patient‐level factors, such as risk awareness, social responsibility, self‐efficacy, and infection experience to deliver targeted education, behavioral support, and risk communication. Incorporating the practical lessons learned from the COVID‐19 response into flexible nursing models will better equip the nursing profession to provide prompt and effective care when faced with infectious disease challenges in the future.

## 6 Conclusion

This study revealed that the unique psychosocial and behavioral factors within different latent subgroups influence the intentions of Korean adults to engage in preventive behaviors for EIDs. Conventional regression analyses identified social responsibility and vaccination behavior as key predictors; however, mixture regression modeling exposed significant variability, highlighting subgroup‐specific pathways influenced by combinations of EID awareness, mask‐wearing behavior, self‐efficacy, and previous COVID‐19. These results highlight the importance of evidence‐based, customized nursing interventions that consider population diversity to improve preventive outcomes. Nurses and public health professionals, by applying lessons learned from the COVID‐19 pandemic, can improve their readiness and response to future infectious disease challenges, providing interventions that are timely and tailored to the distinct motivational profiles of various population subgroups.

## Author Contributions

All authors have agreed on the final version and meet at least one of the following criteria. Study conception and design acquisition‐YHM, CJH; Data collection‐ YHM; Analysis and interpretation of the data, Drafting and critical revision of the manuscript‐ YHM, CJH.

## Funding

This study received no specific grant from any funding agency in the public, commercial, or not‐for‐profit sectors.

## Conflicts of Interest

The authors declare that they have no known competing financial interests or personal relationships that could have appeared to influence the work in this paper.

## IRB Approval Number

Yonsei University Wonju Severance Christian Hospital Institutional Review Board (IRB Approval No. CR322159).

## Data Availability

The data supporting the findings of this study are available from the corresponding author upon reasonable request, in accordance with institutional review board approval and ethical guidelines.
